# Fabrication of Graphene-isolated-Au-nanocrystal Nanostructures for Multimodal Cell Imaging and Photothermal-enhanced Chemotherapy

**DOI:** 10.1038/srep06093

**Published:** 2014-09-02

**Authors:** Xia Bian, Zhi-Ling Song, Yu Qian, Wei Gao, Zhen-Qian Cheng, Long Chen, Hao Liang, Ding Ding, Xiang-Kun Nie, Zhuo Chen, Weihong Tan

**Affiliations:** 1 Molecular Science and Biomedicine Laboratory, State Key Laboratory of Chemo/Bio-Sensing and Chemometrics, College of Chemistry and Chemical Engineering, College of Biology, Collaborative Innovation Center for Chemistry and Molecular Medicine, Hunan University, Changsha, 410082, China; 2 Faculty of Sciences, University of Macau, Av. Padre Tomás Pereira Taipa, Macau, China; 3 Department of Chemistry and Department of Physiology and Functional Genomics, Center for Research at Bio/nano Interface, Shands Cancer Center, UF Genetics Institute and McKnight Brain Institute, University of Florida, Gainesville, Florida 32611-7200, United States

## Abstract

Using nanomaterials to develop multimodal systems has generated cutting-edge biomedical functions. Herein, we develop a simple chemical-vapor-deposition method to fabricate graphene-isolated-Au-nanocrystal (GIAN) nanostructures. A thin layer of graphene is precisely deposited on the surfaces of gold nanocrystals to enable unique capabilities. First, as surface-enhanced-Raman-scattering substrates, GIANs quench background fluorescence and reduce photocarbonization or photobleaching of analytes. Second, GIANs can be used for multimodal cell imaging by both Raman scattering and near-infrared (NIR) two-photon luminescence. Third, GIANs provide a platform for loading anticancer drugs such as doxorubicin (DOX) for therapy. Finally, their NIR absorption properties give GIANs photothermal therapeutic capability in combination with chemotherapy. Controlled release of DOX molecules from GIANs is achieved through NIR heating, significantly reducing the possibility of side effects in chemotherapy. The GIANs have high surface areas and stable thin shells, as well as unique optical and photothermal properties, making them promising nanostructures for biomedical applications.

The use of nanomaterials in biomedicine has allowed researchers to combine multiple functionalities into a single system. Accordingly, such materials as quantum dots^1^,^2^, gold nanoparticles^3^,^4^,^5^, carbon nanotubes^6^,^7^, graphene^8^,^9^, and polymersomes^10^ have all been investigated and have shown promise in a wide range of biomedical applications^11^. Simultaneous diagnostics, therapeutics, and monitoring of response to treatment are realized with such multifunctional nanomaterials and nanostructures, which also offer the potential of both reducing common chemotherapy- or radiation-associated side effects and increasing the effectiveness of therapy^12^,^13^,^14^.

NIR light, in the range of 700–900 nm or 1000–1350 nm, which is the main optical transparent window for biosamples, has been widely used for bioimaging to improve spatial resolution and help localize the region needed for therapy^15^,^16^. Nonlinear two-photon luminescence (TPL)^17^, upconversion fluorescence^18^,^19^ and Raman^20^,^21^,^22^,^23^ imaging have been widely used techniques with NIR excitation. Moreover, because of its easy use and minimal absorbance by skin and tissues, NIR radiation is also an attractive external stimulus to control the toxicity of the therapy through controlled release or enhanced therapeutic efficiency, thereby allowing noninvasive penetration of deep tissues^24^,^25^,^26^,^27^,^28^. Conventional nanomaterial candidates featuring NIR imaging and photothermal properties are generally based on gold nanocages or nanorods^12^,^29^,^30^,^31^,^32^. These materials have strong NIR absorbing capability and can efficiently convert NIR light into heat through photothermal processes. However, they exhibit relatively low thermal stability during prolonged laser irradiation. Therefore, a strategy that improves the thermal stability of gold nanocrystals is essential to further extend their biomedical applications.

On the other hand, for imaging applications, gold nanocrystals are good TPL agents^17^. They are also widely used as substrates for Raman measurements^20^,^21^,^22^,^23^, because of the surface plasmon resonance generated on the gold nanocrystal during laser irradiation, which is known as surface-enhanced Raman scattering, or SERS. In fact, SERS enhancement efficiency is related to nanocrystal morphology and the distance between the Raman signal molecules and the gold substrate. However, molecules directly attached to the bare gold surface can cause photocarbonization of signal molecules or nearby impurity molecules under strong or prolonged laser irradiation^33^. Therefore, accurately controlling the distance between the molecule generating the Raman signal and the gold substrate, as well as maintaining morphology and protecting Au nanocrystals, is important, but challenging.

To address the issue, a chemically inert shell coating around the Au nanoparticle to protect the nanostructure from contacting with the object is probed. Different strategies have been explored, such as coating with a shell of silica or alumina on the Au nanocrystal surfaces^34^,^35^. However, controlling the stability and thickness of the shell is still an issue for the application of such Au nanocrystals. Graphene is a two-dimensional atomic crystal consisting of a single-layer of carbon atoms densely packed into a honeycomb lattice^36^. It is chemically inert and its thickness is relatively easy to control, making graphene a promising shell material for coating the Au nanocrystal^37^,^38^. In addition, graphene has unique NIR absorbing properties and a large surface area, both of which are appealing for loading drug molecules for thermal- and chemotherapy^25^,^26^,^27^,^28^,^39^,^40^. The strong Raman scattering signal of graphene has also made it a useful Raman tag for imaging and measurements^6^,^25^,^26^,^27^,^28^,^41^,^42^,^43^. Therefore, integrating the unique properties of both Au nanocrystals and graphene will offer increased potential for applications in biomedicine. Coating graphene on single AuNP surfaces has already been tried, since such a nanomaterial has interesting and promising properties. However, most of these modifications tend to modify the AuNPs with graphene oxide (GO)^37^. The thickness and amount of GO adsorbing on the AuNP surface is hard to control. And normally it is necessary to reduce the GO into rGO to obtain the pristine and unique properties of the graphene. Since it is difficult to control the quality of the reduction, this step complicates the process. So it is challenging to develop a simple but efficient method to fabricate graphene coated Au nanostructures.

Some researchers also tried using chemical vapor deposition (CVD) to coat the AuNP with graphene, since CVD is commonly used to produce high-purity, high-performance solid materials. However, most of the reports involve coating the AuNP with graphene on silicon substrates^38^. Highly efficient and large scale synthesis is difficult, and graphene layer thickness control is even more challenging. As a novel solution to this problem, we designed a CVD process to encapsulate the Au nanocrystal within a thin layer of graphene and investigated the applications for bioimaging and cancer therapy. The synthesized graphene-isolated-Au-nanocrystals (GIANs) demonstrated excellent stability and robustness against chemical attacks in harsh environments. We also utilized GIANs as Raman enhancement substrates to obtain cleaner vibrational information, free from various metal-molecule interactions. The Raman signals were more stable against photoinduced damage, without compromising the enhancement factor.

The thin graphene shell of GIANs also exhibited strong and unique Raman scattering properties, which were utilized as SERS Raman tags for cell staining and imaging. GIANs were also found to have strong NIR TPL, which was explored as another mode for cell staining and imaging. Moreover, aptamer-functionalized GIANs were developed through π-π interactions to realize targeted cell imaging. The GIAN's graphene shell also proved to be a good substrate for loading the anticancer drug DOX for chemotherapies. The unique NIR absorbance also endowed GIANs with photothermal therapeutic capability, which could be combined with chemotherapy to further improve therapeutic efficiency. Finally, the NIR photothermal effect of GIANs was used to control the release of DOX in a noninvasive manner, thus significantly reducing the side effects of chemotherapy.

## Results

### Synthesis and Characterization of GIANs

GIANs were synthesized in a CVD system. Briefly, HAuCl_4_ metal precursors in aqueous solution were first loaded onto fumed silica powder by impregnation in a methanol solution. The metal-loaded silica was then dried and subjected to methane CVD for graphene shell growth. Once cooled to room temperature, the powdered materials were treated with hydrofluoric acid to dissolve the silica, followed by washing with water to obtain the graphene-encapsulated Au nanocrystal.

**Figure 1:**
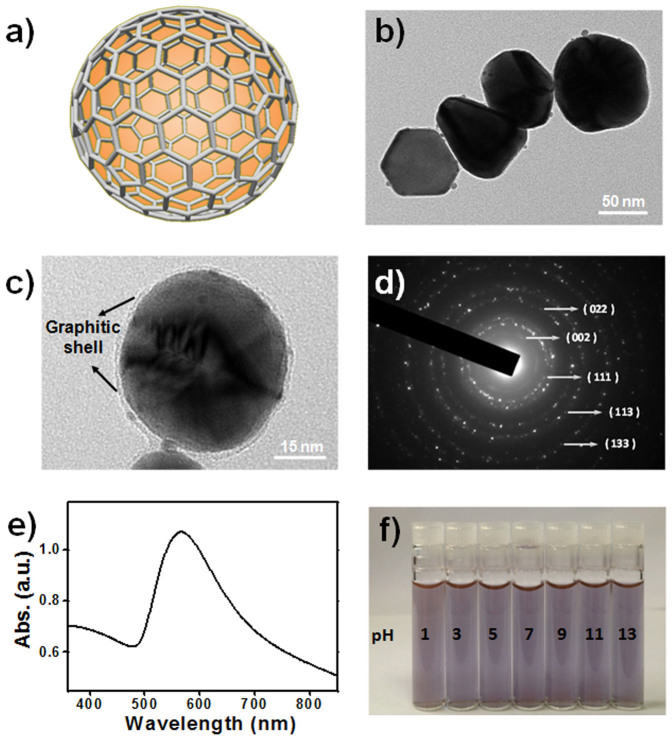
Advanced structural analysis and chemical properties of GIAN. (a) Schematic diagram of GIAN. (b) and (c), TEM images of GIANs. (d) Selected area electron diffraction measurement of GIANs. (e) UV-Vis spectrum of an aqueous GIAN suspension. (f) GIAN suspensions ranging from very acidic pH 3 to alkaline pH 11 conditions, respectively.

The GIANs, synthesized as core-shell nanostructures (Fig. 1a), were characterized by transmission electron microscopy (TEM). As shown in Fig. 1b, TEM imaged GIANs demonstrate a size distribution with an average diameter around 65 nm. Further higher resolution TEM images, as presented in Fig. 1c, show the GIAN as an Au nanocrystal core isolated with a shell structure of a thin layer of graphene (see the Supplementary, Fig. S1 for more TEM characterizations). Compared to other silica or alumina isolation techniques, the shell thickness of graphene synthesized here is uniform and achieved in a much more controllable manner as a result of the ultrathin and stable single-atom thickness of the graphene layer, a unique feature of GIANs.

Selected area electron diffraction (SAED) was also utilized to characterize the GIANs' crystal structure (Fig. 1d). The Au-(111), -(022), -(113), and -(133) facets presented a face-centered cubic structure of the GIAN crystal core, while the (002) facet was assigned to the hexagonal crystalline graphite shell of the GIAN. Dynamic light scattering (DLS) measurement further confirmed the average GIAN size (Supplementary, Fig. S2), and ζ-potential measurements showed that the GIANs were uncharged, as a result of the pristine properties of the graphene shells (Supplementary, Fig. S3).

The as-prepared GIANs were not water soluble because of the hydrophobic carbon surface. Polyoxyethylene stearyl ether molecules were utilized to noncovalently functionalize the graphene shells. The alkyl chains of the PEG molecules anchor on the GIAN surface through hydrophobic-hydrophobic interactions and solubilize the GIAN in water. Fig. 1e shows the UV-Vis spectrum of the aqueous GIAN suspension. Consistent with GIAN size, strong plasma absorbance around 570 nm was observed. GIANs exhibited superior stability from the excellent robustness of the graphitic shell which could resist chemical attack in both gaseous and liquid environments. The well-functionalized GIANs were found to be very stable under extreme pH environments and high salt conditions. As shown in Fig. 1f, GIANs are stable in solution, ranging from very acidic pH 3 to alkaline pH 11 conditions.

### SERS Detection and Raman Imaging with GIANs

**Figure 2:**
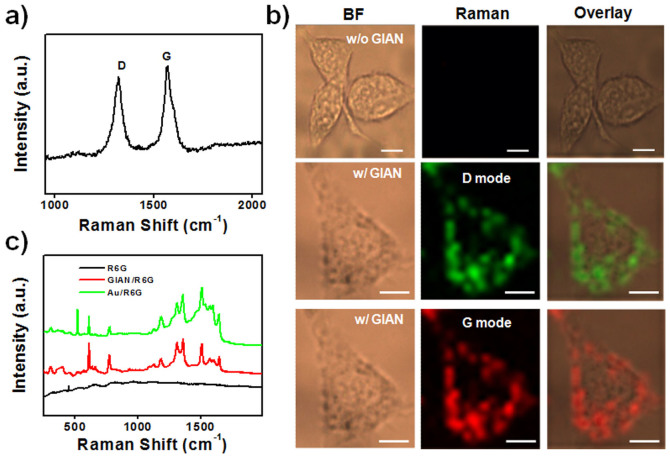
Raman properties of GIANs. (a) Raman spectrum (excitation at 632 nm) of GIANs showing the G and D bands of graphitic carbon. (b) Raman imaging of MCF-7 cells with and without GIAN staining. BF: bright field, scale bar: 10 μm. (c) Raman spectra of R6G molecules, with and without GIAN, and with Au nanoparticles, respectively.

GIANs have unique Raman scattering properties. Two prominent bands can be observed around 1325 and 1595 cm^−1^ (Fig. 2a), corresponding to the D and G Raman vibrational modes of graphitic carbon shells, respectively. The high intensity (relative to the G peak) Raman D peak reflects the high strain of the graphite shells as a result of the deformation of the flattened graphene encapsulating the Au nanocrystals. The GIAN has a strong and simple resonance Raman signature and can be utilized as a good Raman tag for cell imaging. Compared to organic Raman active molecules, GIANs, as graphitic nanomaterials, have enormous Raman scattering cross sections (~10^−21^ cm^2^ sr^−1^ molecule^−1^) and are much more stable based on their inorganic structure^6^.

Fig. 2b shows the Raman images of MCF-7 breast cancer cells stained with GIANs. Both the D and G modes can be used to image cells. The gold nanocrystal core significantly enhanced the Raman signal of the graphene shell, making the MCF-7 cells light up clearly under laser excitation. All the Raman signals were distributed throughout the cellular cytoplasm, while no signal was associated with the nuclei, indicating the distribution of GIANs in the cell. Compared to fluorescence imaging, the Raman signal in MCF-7 cells had good imaging resolution, as indicated by the narrow full width at half maximum (FWHM) of the Raman scattering peak and the stable signal from the unbleached GIAN Raman signal. This represents a potentially useful tool for monitoring endocytosis processes of GIANs in the cells.

The GIAN is also a good enhancement substrate for the detection of small molecules. It has a high surface area for adsorption of small molecules. The main virtue of such a graphene shell-isolated substrate lies in its higher detection sensitivity and vast practical applications. By using GIANs, we can obtain more meaningful surface Raman signals, because the chemically inert thin shell prevents distortions due to direct interaction between the probed adsorbates and bare Au nanoparticles, while still maintaining a significant Raman enhancement effect^33^,^34^,^35^. Rhodamine 6G (R6G) dye was used as a model molecule for detection and determination of the SERS effect (Fig. 2c). GIANs significantly enhanced the R6G Raman signal, with a signal amplification factor in excess of 100 when GIANs were added to the analyte solution. GIANs also quenched the R6G background fluorescence through the fluorescence resonance energy transfer (FRET) process, in turn improving the R6G Raman signal-to-noise ratio. Further improvement of the enhancement factor could be achieved through designing the GIAN structure and optimizing the measurement method, which we will investigate in the future. We further compared the bare gold nanoparticles with GIANs and observed that GIANs also substantially reduced the photocarbonization or photobleaching of the R6G analytes. It is speculated that the presence of graphene isolation suppresses the catalytic activity of the gold nanocrystal core and prevents R6G molecules, as well as other adsorbates from either the solution or the environment, from having direct contact with the gold core, which would result in photocarbonization^33^.

### Two-photon Luminescence Imaging with GIANs

The GIAN also has unique TPL properties. Because of the autofluorescence background problem of one-photon bioimaging, the use of two- or multiphoton imaging has recently attracted attention. Two-photon excitation-induced bioimaging has many unique advantages. Owing to a square/cubic or higher dependence of multiphoton absorption on laser intensity, the sample region outside the beam focus cannot be excited, thus reducing the potential for photobleaching of the sample signal. The nonlinear excitation mode also helps to improve the spatial resolution of imaging, as only the site where the laser beam is focused can be efficiently excited^44^. Two-photon excitation wavelengths are usually in the NIR range, which is the main optical transparent window for biosamples, as water has very small light absorption in this range.

**Figure 3:**
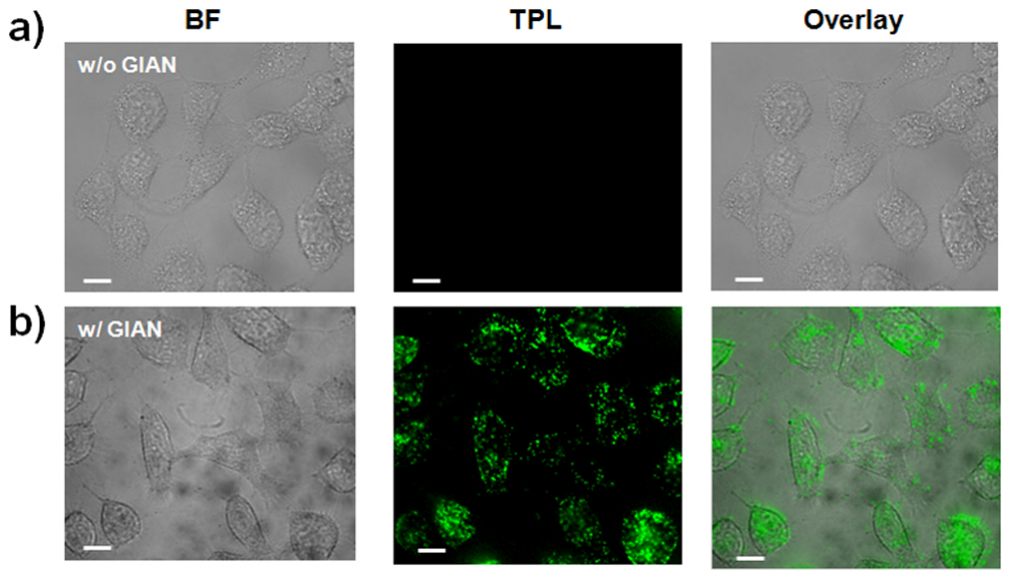
TPL confocal images of MCF-7 cells without (a) and with (b) GIAN staining. Scale bar: 10 μm.

The TPL properties of GIANs were investigated for cell imaging. A wavelength of 850 nm was utilized as the excitation laser, while emission was collected with a two-photon microscope. As shown in Fig. 3, MCF-7 cancer cells incubated with GIANs demonstrated bright TPL signals. However, in the absence of GIAN incubation, no obvious TPL signal of control MCF-7 cells was observed (Fig. 3a), in agreement with the low NIR light absorption of biosamples. GIANs demonstrated good biocompatibility, and bright TPL signals were observed in the MCF-7 cells stained with GIANs (Fig. 3b). Consistent with the Raman images, the TPL signals were distributed throughout the cellular cytoplasm. Both the Au nanocrystal core and graphene outer layer of the GIANs are believed to contribute to the TPL signals (Supplementary, Fig. S4). The GIANs could exhibit TPL signals from the surface plasmon resonance of the Au nanocrystal core^44^,^45^, as well as from the two-photon absorption of the sp^3^ hybridization state of the graphene shell^46^,^47^,^48^.

### Aptamer Functionalization of GIANs for Targeted Imaging

**Figure 4:**
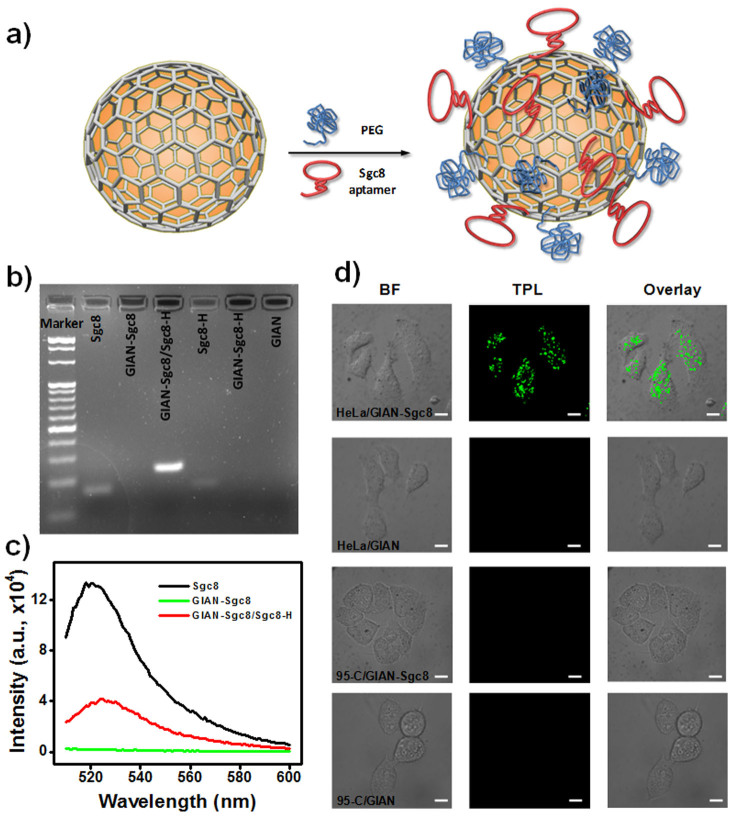
Targeted cell imaging with GIANs. (a) Schematic illustration of Sgc8 aptamer-functionalized GIAN. (b) Gel electrophoresis characterization of aptamer-functionalized GIANs. (c) Fluorescence characterization of aptamer-functionalized GIANs. (d) TPL confocal images of HeLa and 95-C cells incubated with GIAN and GIAN-Sgc8. BF: bright field, scale bar: 10 μm.

The GIAN graphene shell is an ideal platform for biomolecule functionalization. In particular, we functionalized GIANs with Sgc8 aptamers, which selectively bind to protein tyrosine kinase 7 (PTK7) cell membrane proteins, to investigate the targeted imaging capability of GIANs^49^. Single-stranded nucleic acids are known to adsorb on a graphene surface through strong π-π interactions, thereby providing a simple and efficient way to functionalize graphitic nanomaterials^50^,^51^,^52^. Fig. 4a schematically illustrates Sgc8 aptamer-functionalized GIANs. We designed the Sgc8 aptamer into a hairpin structure with an eight-A-base tail, which helps the aptamer to anchor on the graphene shell, since the A base has strong binding affinity with the graphene surface^53^, while, as described below, aptamer activity is maintained.

The efficiency of aptamer functionalization was investigated by gel electrophoresis. As shown in Fig. 4b, beside the marker lane, the next two columns of the gel were Sgc8 aptamer and GIAN-Sgc8 complexes. No band was observed in the GIAN-Sgc8 lane, indicating the absence of free Sgc8 aptamer. We also added Sgc8-H, the complementary DNA strand of Sgc8, to the GIAN-Sgc8 solution. A new band was observed, indicating formation of double-stranded DNA (dsDNA) of the Sgc8 aptamer. This occurred because dsDNA has weak interaction with the graphene shell and could therefore be separated during electrophoresis. Gels were also obtained for Sgc8-H, GIAN-Sgc8-H complexes and GIAN alone, and the results all demonstrated good functionalization of the GIAN.

We further identified the functionalization of GIANs using FAM-modified Sgc8 aptamers. Graphene has been demonstrated as a good quencher of nearby fluorescence dyes^54^,^55^. As shown in Fig. 4c, the fluorescence signal of GIAN-Sgc8 was significantly reduced compared to Sgc8 aptamer alone. After adding the Sgc8-H aptamer to the Sgc8 solution, obvious fluorescence recovery was observed, indicating good Sgc8 functionalization on GIANs. The GIAN-Sgc8 complex was then utilized for targeted cell TPL imaging. HeLa cells and 95-C lung cancer cells were used for imaging since they showed different binding affinities to Sgc8 aptamers (Supplementary, Fig. S5). Fig. 4d shows the TPL images of the targeting tests. The cells were incubated with GIAN and GIAN-Sgc8 at 4°C for two hours, respectively. Only the HeLa cells, which had higher binding affinities with Sgc8 aptamers, showed a positive TPL signal. Thus, GIAN-Sgc8 demonstrated good targeted imaging capabilities, indicating efficient functionalization of the Sgc8 aptamer on the GIAN surface and, hence, its potential in biomedical applications.

### NIR Photothermal Therapy with GIANs

**Figure 5:**
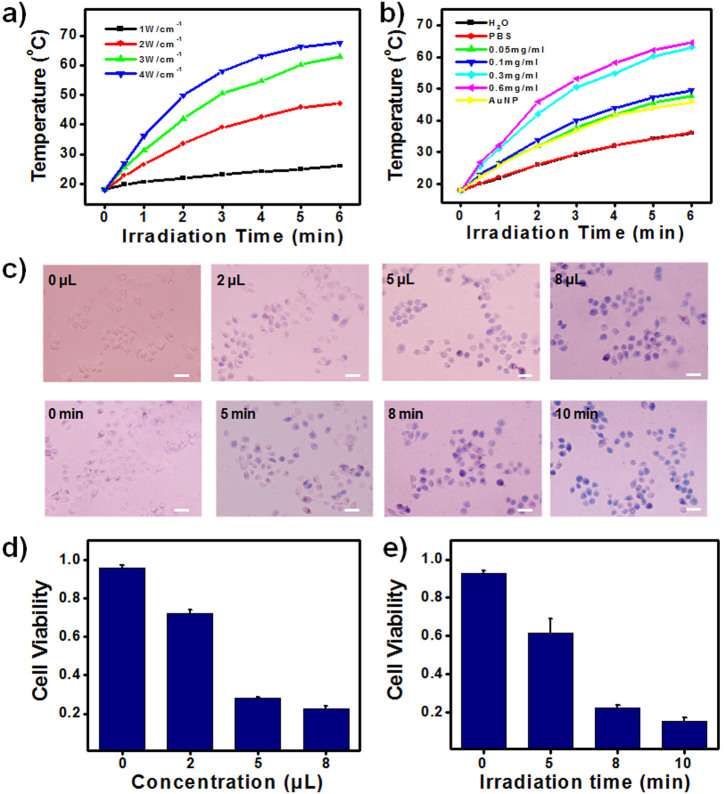
NIR photothermal effect of GIANs. (a) Heating curve of 0.3 mg/mL GIAN solution using different power densities of an 808 nm NIR laser. (b) Heating curve of GIANs solution with different concentrations under 3 W/cm^2^ laser irradiation. (c) Bright field microscopy images of trypan blue-stained MCF-7 cells after different NIR photothermal treatments. Scale bar: 50 μm. (d) and (e), relative cell viability after treatment with different GIAN concentrations and different 808 nm laser irradiation times, respectively.

Graphitic nanomaterials have strong absorbance in the NIR region, making them suitable for NIR photothermal therapies^25^,^26^,^27^,^28^,^39^,^40^. Au nanocrystals also have strong light absorbing capability, which makes them ideal for wide use in the photothermal therapy of cancers^29^,^30^,^31^,^32^. Combining the unique properties of graphitic and Au nanomaterials, GIANs demonstrated excellent photothermal heating capability. Fig. 5a shows the heating curve of 0.3 mg/mL GIANs solution under different power densities from 1 W/cm^2^ to 4 W/cm^2^ of an 808 nm NIR laser. After 8 minutes of 2 W/cm^2^ laser irradiation, the solution temperature increased to around 30°C, which is much higher than that of the 1 W/cm^2^ laser irradiation. We also investigated the concentration effect of photothermal heating (Fig. 5b). Upon increasing the concentration of GIANs, the solution temperature increased after 8 minutes of 3 W/cm^2^ laser irradiation. Au nanoparticles (0.3 mg/mL) were also used to compare heating efficiency, and a temperature increase similar to that of the 0.05 mg/mL GIAN solution was observed. The photothermal heating of GIAN is very stable, even under prolonged laser irradiation time, because of the highly stable graphene-isolated shells on the GIANs (Supplementary, Fig. S6).

MCF-7 cells were then utilized for *in vitro* photothermal treatment. The GIAN concentration and laser irradiation time were optimized to determine photothermal killing efficiency. Fig. 5c shows the bright field microscopy images of trypan blue-stained MCF-7 cells after different treatments. Without GIANs, no obvious cell death was observed after 8 minutes of 808 nm laser irradiation. Also, the addition of 8 μL 0.2 mg/mL GIANs into the cell culture without the laser irradiation did not result in any observable cell death, indicating the good biocompatibility of GIANs. Fig. 5d and 5e show relative cell viability for different GIAN concentrations and different 808 nm laser irradiation times, respectively. Increasing GIAN concentration or laser irradiation time could efficiently improve the photothermal destruction effect. Thus, with the addition of 8 μL 0.2 mg/mL GIAN solution and irradiation with a 808 nm laser over 8 minutes, more than 80% of the cells were found dead, indicating the efficiency of GIAN as a good material for NIR photothermal therapies.

### NIR Photothermal Enhanced Chemotherapy with GIANs

**Figure 6:**
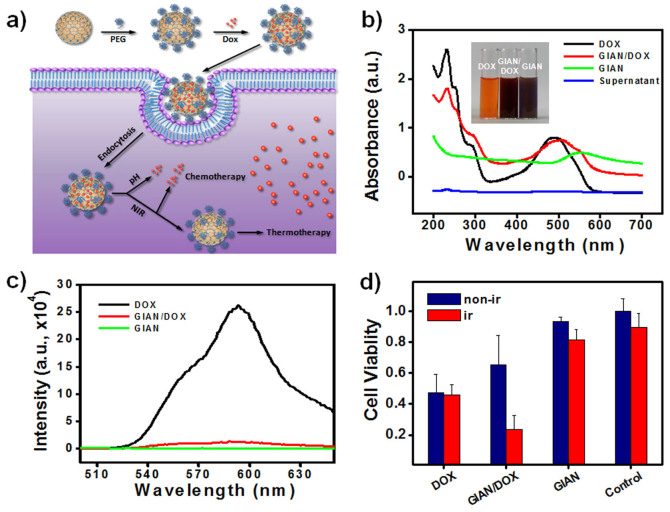
Photothermal enhanced chemotherapy with GIANs. (a) Schematic illustration of NIR photothermal enhanced chemotherapy mechanism of GIAN/DOX complexes. (b) UV-Vis characterization of the DOX-loaded GIANs. Inset: digital photo of the DOX, GIAN, and GIAN/DOX solutions. (c) Fluorescence spectroscopy characterization of the DOX loading efficiency. (d) Cell viability of MCF-7 cells with and without NIR laser irradiation after incubation with free DOX, GIAN, and GIAN/DOX, respectively.

Chemotherapy is another common therapy for cancer treatments. Combining chemotherapy with photothermal therapy would significantly enhance therapeutic efficiency. DOX is a widely used anthracycline antibiotic to treat various cancers^56^. GIAN proved to be a good substrate for loading DOX via π-π stacking (Fig. 6a). Moreover, GIAN/DOX complexes constitute a good platform for enhancing therapeutic efficiency through combining chemotherapy and hyperthermia. Fig. 6a is a schematic illustration of the NIR photothermal-enhanced chemotherapy mechanism. After GIAN/DOX endocytosing into the cancer cells, the DOX was partially released from the GIAN surface by decreasing the pH. As described below, the NIR photothermal effect of the GIAN could be further utilized to control the unloading of the chemotherapeutic DOX molecules.

DOX loading was first investigated. Fig. 6b shows the UV-Vis characterization of DOX-loaded GIANs. The peak on the UV-Vis spectrum around 490 nm corresponds to the DOX molecules, while GIAN has an absorbance peak around 570 nm. The conjugated GIAN/DOX solution was washed several times. The supernatant and precipitate were both collected and characterized by UV-Vis. The final supernatant had no peak in the UV-Vis spectrum, while the washed GIAN/DOX had a broad absorbance from 400 to 600 nm. Two obvious peaks around 490 nm and 570 nm indicate conjugation of DOX on the GIAN surface. The inset in Fig. 6b is a digital photo of DOX, GIAN/DOX and GIAN solution. The GIAN/DOX suspension was very stable and could be stored for more than one month before use. DOX loading efficiency was further explored by fluorescence spectroscopy (Fig. 6c). After DOX was loaded on the GIANs, the fluorescence of the DOX was quenched by FRET, indicating the close attachment of DOX on the GIAN surface.

The prepared GIAN/DOX complexes were then used for cytotoxicity tests *via* the methyl thiazolyl tetrazolium (MTT) assay with MCF-7 cells. DOX, GIAN/DOX and GIAN were incubated with MCF-7 cells to compare therapeutic efficiency, respectively. As shown in Fig. 6d, the toxicity of the GIAN, GIAN/DOX and DOX in MCF-7 cells increased successively. This indicated good biocompatibility of the GIANs and the relatively slow release of DOX from the GIAN/DOX samples by the lower pH inside the endosome. However, when all the MCF-7 cell samples were irradiated with the 808 nm laser for 8 minutes, the toxicity order was changed. No obvious differences in cell death were observed for the DOX, GIAN and control MCF-7 cell samples with and without the laser irradiation. On the other hand, for the GIAN/DOX sample, after 808 nm laser irradiation, cell death significantly increased and was even higher than that of the DOX-only samples, demonstrating the efficient photothermal enhancement of the chemotherapeutic effect. The controllable drug release with NIR laser irradiation can significantly reduce the side effects of chemotherapeutic applications, making the GIAN a promising platform for future clinical applications.

## Discussion

We have developed a biocompatible nanocrystal system for combined drug delivery, imaging, and NIR-induced hyperthermia. A GIAN is synthesized by the CVD method. A very thin layer of graphene is grown on the Au core without causing aggregation of the Au nanocrystals. The GIAN nanocrystal exhibits superior stability based on the excellent robustness of the graphitic shell.

The GIANs are used as Raman enhancement substrates, and they significantly enhance the Raman signal of analytes, such as R6G, in solution. GIAN quenches the R6G background fluorescence and also prevents its direct contact with the gold core, thus eliminating the concern of photocarbonization, leading to an improved R6G Raman signal-to-noise ratio. The GIAN itself also has strong and unique Raman scattering properties and is demonstrated to be a good Raman tag for cell staining and SERS Raman imaging. The GIAN also proves to be a good TPL agent for still another mode of cell imaging with an NIR laser. Moreover, the GIAN is found to be a good platform for biomolecule functionalization. Targeted cell imaging was realized by designing aptamer-functionalized GIANs via strong π-π interaction.

The GIAN is also identified to be a good material for NIR photothermal therapies. With 808-nm laser irradiation, cancer cells stained with GIANs can be efficiently killed through NIR-induced hyperthermia. The anticancer drug DOX is also loaded on the GIAN platform for chemotherapy. The GIAN/DOX complex is found to significantly enhance therapeutic efficiency by combining chemotherapy and hyperthermia. In addition, the controlled release of DOX molecules loaded on GIANs is accomplished through NIR thermal heating, which could significantly reduce the side effects of chemotherapeutic applications. Photothermally enhanced drug delivery enabled by the highly integrated GIAN/DOX system can offer a new method for highly effective drug delivery and cancer imaging, while reducing systemic drug-related toxicity, thus bringing a promising tool to the practice of clinical medicine.

## Methods

### Raman Characterization and Imaging with GIANs

A 10 mM stock solution of the R6G dye was prepared in water and further diluted to the desired concentrations. The SERS samples were obtained by adding 10 μL 100 μM R6G solution to 15 μL colloidal samples. For each Raman spectrum, more than five measurements were conducted in a Renishaw Raman imaging microscope system with a 633 nm excitation laser. For Raman imaging, all human breast cancer cells (MCF-7) were incubated with GIAN solutions for 2 h at 37°C. The final GIAN concentration in these incubation solutions was 2.5 × 10^−3^ mg/mL. After washing with Dulbecco's phosphate buffered saline (DPBS, Gibco) three times, cells were imaged under a Raman confocal microscope with a 633 nm laser. Each Raman mapping image contained at least 50 × 50 spectra.

### Aptamer Functionalization, Characterization and Targeted Cell Imaging

Sgc8 was prepared as a 1 μM solution in 20 mM Tris-HCl buffer (pH 7.4, containing 100 mM NaCl and 2 mM MgCl_2_) and mixed with 0.09 mg/mL GIAN for 30 min prior to the addition of Sgc8-H. Then Sgc8-H was added to the prepared GIAN-Sgc8 solution, and about 15 min was allowed for hybridization at 37°C. Finally, the fluorescence of Sgc8-FAM, GIAN-Sgc8-FAM and GIAN-Sgc8-FAM/Sgc8-H was detected with a fluorescence analyzer, and aptamer functionalization efficiency was investigated with gel electrophoresis. After the samples were loaded, a 1 × TBE buffer as running solution was added into the outer buffer reservoir until the liquid level just covered the top surface of the gel. A 20 bp DNA Ladder (TaKaRa, China) was added in the first lane as the molecular weight size marker. Ethidium bromide dyes (Dingguo Changsheng Ltd., China) were added to stain the nucleic acids. The electrophoresis was run at a constant bias of 120 V. For targeted cell imaging, HeLa and 95-C cells were cultured in a 30-mm glass bottom dish with 2 mL media for one day. After cells were washed, GIAN and GIAN-Sgc8 were added to the cell wells, and the cells were incubated for 2 hours at 4°C. The cells were then carefully rinsed two times, followed by analysis using a FV1000-X81 confocal microscope (Olympus) equipped with a 60× objective with an 850 nm excitation laser.

### NIR Photothermal Treatment of Cancer Cells

To determine the effect of GIAN concentration on the photothermal heating effect, a series of solutions of GIAN in PBS (pH 7.4) with different GIAN concentrations from 0.05 to 0.6 mg/mL were irradiated by NIR laser (wavelength, 808 nm; power density, 3 W/cm^2^; Kaisite Electronic Equipment Co., Ltd., Beijing, China) for different times. To determine the effect of NIR power density, the 0.3 mg/mL GIAN solution was irradiated using different power densities, while the temperature was monitored by a thermometer. AuNP normalized to a GIAN concentration at 0.3 mg/mL, together with PBS (pH 7.4) and water, were used as controls. For photothermal cell therapy, MCF-7 cells were precultured for 24 h, and then GIAN was added with a final concentration of 3.5 × 10^−3^ mg/mL. After incubation for 2 h at 37°C, cells were washed with DPBS to remove excess GIANs. Then an 808 nm NIR laser was used to irradiate MCF-7 cells at a power density of 2 W/cm^2^ at different times and with various GIAN concentrations. After staining with 0.4% Trypan blue solution (Sigma Inc.) for 5 min, microscopic images of the cells were obtained using an epifluorescence microscope (Olympus IX71, 40×, 0.65 N.A. objective).

### Cell Viability Test of Photothermal Enhanced Chemotherapy

The standard cell viability assay using 3- (4,5-dimethylthiazol-2-yl)-2,5-diphenyltetrazolium bromide (MTT, Sigma Inc.) was carried out to determine MCF-7 cell viability relative to the control cells incubated with the same volume of DPBS. First, MCF-7 cells were cultured in 96-well microplates with 100 μL of media for 18 hours. DOX, GIAN and GIAN/DOX were added to separate wells and cultured for 2 h, respectively. Then the cells were washed with DPBS and added to 100 μL of culture media. For thermal therapy, corresponding wells were irradiated with an 808 nm NIR laser (3 W/cm^2^) for 5 min. The culture media were then replaced with fresh media and cultured for a further 48 h. Then 20 μL of MTT solution was added to each well and incubated for another 4 h at 37°C. Upon termination of culturing, the medium was carefully aspirated by adding 150 μL of dimethyl sulfoxide to each well and setting shaker speed oscillation to 10 min to fully dissolve the crystals. After that, the absorbance was measured with a microplate reader (BioTek) at 450 nm. The viability of treated cell wells was expressed as a percentage of the viability of unexposed wells.

## Author Contributions

Z.C., W.T. and X.B. conceived the project, designed the experiments and wrote the paper. X.B., Z.L.S. and Y.Q. conducted all the experiments. W.G., Z.Q.C. and X.K.N. assisted with the GIAN synthesis. D.D. assisted with the TEM characterization. L.C. and H.L. contributed to the Raman imaging and data analysis.

## Supplementary Material

Supplementary Information Supplementary Information
