# Supplementary material for: Fabrication of Graphene-isolated-Au-nanocrystal Nanostructures for Multimodal Cell Imaging and Photothermal-enhanced Chemotherapy
Source: Sci Rep. 2014 Sep 2;4:6093. doi: 10.1038/srep06093 (PMC4151100; doi:10.1038/srep06093)
Supplement: Supplementary Information [file srep06093-s1.pdf]

Supporting Information

Fabrication of Graphene-isolated-Au-nanocrystal Nanostructures for Multimodal Cell Imaging and Photothermal-enhanced Chemotherapy

Xia Bian,[1] Zhi-Ling Song,[1] Yu Qian,[1] Wei Gao,[1] Zhen-Qian Cheng,[1] Long Chen,[2] Hao Liang,[1] Ding Ding,[1] Xiang-Kun Nie,[1] Zhuo Chen,[*,1] and Weihong Tan[*,1,3]

[1] Molecular Science and Biomedicine Laboratory, State Key Laboratory of Chemo/Bio-Sensing and Chemometrics, College of Chemistry and Chemical Engineering, College of Biology, Collaborative Innovation Center for Chemistry and Molecular Medicine, Hunan University, Changsha, 410082, China

[2] Faculty of Sciences, University of Macau, Av. Padre Tomás Pereira Taipa, Macau, China

[3] Department of Chemistry and Department of Physiology and Functional Genomics, Center for Research at Bio/nano Interface, Shands Cancer Center, UF Genetics Institute and McKnight Brain Institute, University of Florida, Gainesville, Florida 32611-7200, United States

*e-mail: zhuochen@hnu.edu.cn, tan@chem.ufl.edu; phone: 352 846 2410

Experimental details

Reagents. Anhydrous chloroauric acid ($HAuCl_4$) and R6G were purchased from Aladdin. Polyoxyethylene (100) stearyl ether ($(C_2H_4O)nC_{18}H_{38}O$) (average Mn ~4670) was purchased from Sigma Aldrich. Trypan Blue Dye (0.4%) was purchased from Bio-Rad. All DNA sequences were synthesized by Shanghai Biotech (China). Sequences of oligonucleotide probes used in this work are as follows: Sgc8: 5-AA AAA AAA ATC TAA CTG CTG CGC CGC CGG GAA AAT ACT GTA CGG TTA GA-3; Sgc8-FAM: 5-AA AAA AAA ATC TAA CTG CTG

CGC CGC CGG GAA AAT ACT GTA CGG TTA GA-FAM-3; Sgc8-H: 5-TC TAA CCG TAC AGT ATT TTC CCG GCG GCG CAG CAG TTA GAT TTT TTT TT-3. Tris-HCl buffer solutions (20 mM Tris, 100 mM NaCl, and 2 mM $MgCl_2$, pH 7.4) were used as working solutions.

DOX was purchased from Hisun Pharmaceutical (Zhejiang, China). RPMI-1640 medium, penicillin streptomycin solution and fetal bovine serum were obtained from Invitrogen. MTT was purchased from Beyotime (China). Gold nanoparticles (40 nm) were purchased from Shanghai JieYi Biotechnology. The ultrapure water used was from a Milli-Q Integral System. All other chemical reagents were analytical grade and used without further purification.

GIAN synthesis. GIAN was produced in a CVD system. First, fumed silica (1.00 g, Aladdin) was impregnated with anhydrous chloroauric acid (1%，29.65mL) in methanol and sonicated for 2 h. Then the methanol was removed, the mixture was dried at 80 ℃, and the powder was ground. Typically, 0.50 g of the powder was used for methane CVD in a tube furnace. The sample grew with a methane flow of 150 $cm^3 min^{-1}$ for 10 minutes. After growth, the sample was etched with 10% HF in H_2O (80%) and ethanol (10%) to dissolve the silica. The GIAN solid product was then washed thoroughly and collected through centrifugation. Hollow graphitic nanocapsules were obtained from a core-shell magnetic graphitic nanomaterial (MG). MG was synthesized with the CVD system as reported previously which was similar to GIANs. The as-prepared MG was then treated with a solution of sulfuric and nitric acid to polish for 4 h, followed by solubilization in water. Excess MGs were removed by an external magnet. The HGNs were collected through centrifugation and washed thoroughly with ultrapure water.

Cell culture. Human breast cancer cells (MCF-7), HeLa cells and 95-C cells were cultured at 37 ℃ in RPMI 1640 medium supplemented with 10% premium fetal bovine serum (FBS) and 1% penicillin /streptomycin in a 5% CO_2 environment.

Two-photon luminscence imaging of cells. MCF-7 cells were seeded in a 30-mm glass dish and incubated for 24 h. After removing cell medium, cells were incubated with GIAN in 2 mL DPBS at 37 ℃ for 2h. Then cells were washed three

times with DPBS, followed by analysis using the FV1000-X81 confocal microscope (Olympus) equipped with a 10x objective and a 60x objective with an excitation laser wavelength of 850nm.

DOX loading on GIANs. DOX loading was achieved by simply mixing aqueous DOX solution and 0.3 mg/mL GIANs. The loading was performed with 3.3 mM DOX PBS solution, adjusting the pH to 8.5-9 by using 0.5 M carbonate buffer. Samples were incubated for 14 hours at room temperature with gentle agitation. After the incubation, excess DOX was removed through centrifugation until no DOX was detected in the supernatant. The concentration of DOX was determined by UV-Vis using a DOX extinction coefficient of 10500 $M^{-1}cm^{-1}$ at 490 nm. All UV-Vis measurements were conducted using a UV-2450 UV-Vis spectrophotometer (Shimadzu).

Characterization. We characterized GIANs with transmission electron microscopy (TEM, JEOL 3010, operated at 120 to 200 kV), Selected area electron diffraction (SAED) and Raman spectroscope (Renishaw, InVia reflex, UK). The UV-Vis spectra were measured on the UV-2450 spectrophotometer (Shimadzu). All fluorescence measurements were carried out on a Fluoromax-4 spectrofluorometer (HORIBA JobinYvon, Edison, NJ). The detection of electrophoresis gels was performed by the Bio-Rad ChemiDoc XRS System.

Characterization of GIANs with transmission electron microscopy

Transmission electron microscopy was applied to characterize GIAN morphology and size distribution. Additional TEM images are shown in Fig. S1. Several layers of shells have grown out of the Au core and isolated the Au nanocrystal. The distance between the two shell layers was observed around 0.34 nm in the high resolution TEM (Fig. S1a), which further confirmed the graphene encapsulated Au nanocrystal structure.

Dynamic light scattering characterization of GIAN

The hydrodynamic diameters of the MGs under investigation were measured

using a Zetasizer Nano ZS90 DLS system equipped with a red (633 nm) laser and an Avalanche photodiode detector (APD) (quantum efficiency > 50% at 633 nm) (Malvern Instruments Ltd., Worcestershire, England). DLS measurements were performed at room temperature at a fixed scattering angle of 90 °. Fig. S2 shows the size distribution of the suspended GIAN. The average size was around 70 nm, which agreed with the size measured from TEM. All size distributions reported here were based on number counting. The average particle size was obtained using a non-negative least squares (NNLS) analysis method. For each sample, two DLS measurements were conducted with a fixed 10 runs, and each run lasted 10 s.

ζ-Potential measurement of GIAN

ζ-Potential measurements were performed in water. The measurements were carried out at room temperature on the ZetaSizer Nano ZS90 equipped with MPT-2 Autotitrator and 4 mW He–Ne Laser (λ_0 = 633 nm) using the Laser Doppler Electrophoresis technique. The ζ-potential was calculated by Dispersion Technology software provided by Malvern according to Smoluchowski approximation in an automatic mode. Fig. S3 shows the ζ-potential curves of the GIAN water solution. The GIANs, which were neutrally charged, showed good quality and very few graphene shell defects.

Two-photon luminescence characterization of GIAN

Two-photon microscopy was applied to characterize the efficient staining of GIANs on MCF-7 cells. Additionl TPL images are shown in Fig. S4. GIANs were incubated with the MCF-7 cells for 4 hours before TPL imaging. To identify the contribution of Au nanocrystal core and isolation graphene shell to TPL imaging, we incubated GIANs (Fig. S4a), Au nanoparticles (Fig. S4b) and hollow carbon capsulates (Fig. S4c) with the MCF-7 cells and they all showed the capability for TPL imaging. Thus, both the Au nanocrystal core and graphene outer layer of the GIANs were believed to contribute to the TPL signals.

Flow cytometry characterization of the selectivity of Sgc8 aptamer

To identify the targeting capability of the Sgc8 aptamer, flow cytometry measurements were performed. All the samples were analyzed in a FACScan flow cytometer (Becton Dickinson). The Sgc8 aptamers were labeled with FAM, an organic dye. A green laser at 494 nm was used as the excitation source. Samples containing HeLa and 95-C cells with a concentration of 10^6 cells/mL were incubated with the desired concentrations of Sgc8 at 37 ℃ in a 200 μL volume of binding buffer for 30 min. The cells were centrifuged, washed three times with 200 μL of washing buffer, redispersed in 200 μL of binding buffer, and subjected to flow cytometry analysis by counting 10000 events. Fig. S5 shows the flow cytometry analysis of the Sgc8 aptamer binding to different cells. It was found that Sgc8 aptamer displayed much stronger binding affinity toward HeLa cells than 95-C cells. This could have been caused by the increase of protein tyrosine kinase 7, the binding protein of Sgc8 aptamer expressed on HeLa cells.

Photostability of GIAN under NIR laser irradiation

The photostability of GIAN was investigated with longer laser irradiation time. No obvious color change of the 0.1 mg/mL GIAN solution was observed after 1 hour of 808 nm laser (2 w/cm^2) irradiation, as shown in Fig. S6 inset. The UV-Vis spectra further confirm the stability of the GIANs. The absorbance of GIANs around 550 nm was found to be almost the same with or without laser irradiation (Fig. S6). The unique stability of the graphene shell of the GIAN was believed to help constrict the Au nanocrystal core inside and prevent the morphology from changing under laser irradiation. The high photostability makes GIAN a promising material for further clinical applications.

Figure S1

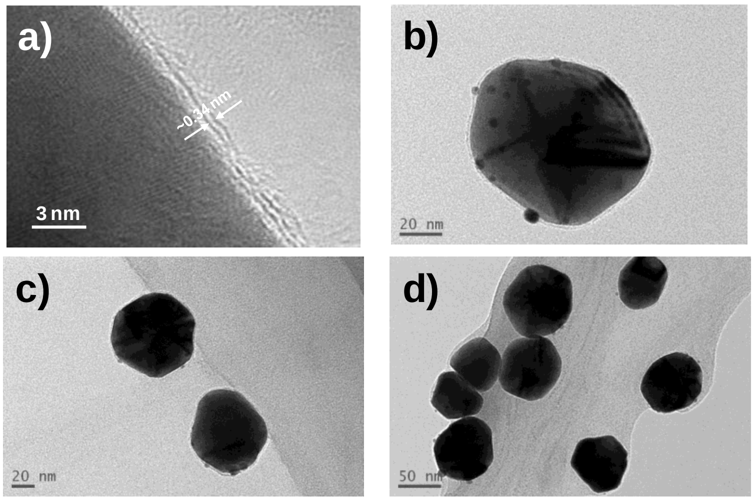

Figure S1. Transmission electron microscopy characterization of GIANs.

Figure S2

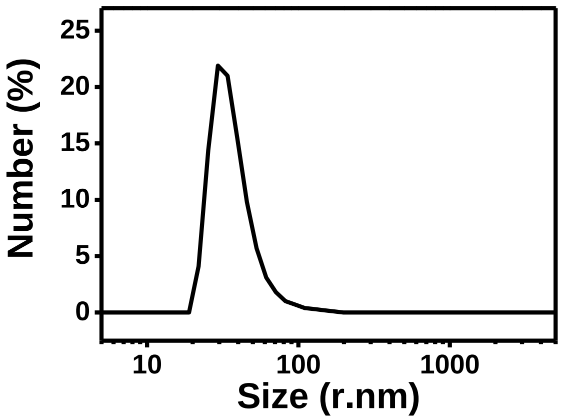

Figure S2. DLS characterization of the size distribution of the suspended GIANs.

Figure S3

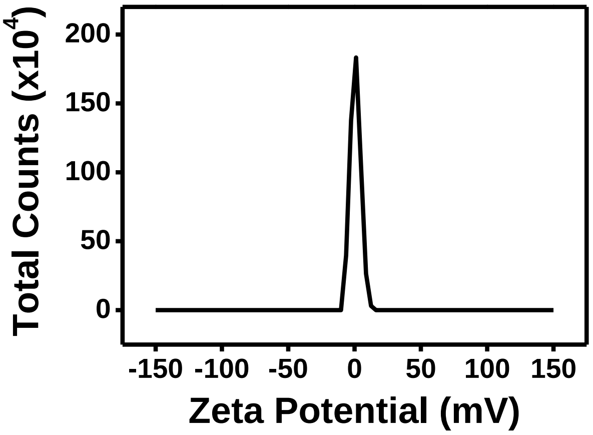

Figure S3. ζ-potential curves of the GIAN suspension measured at room temperature on the ZetaSizer Nano ZS90.

Figure S4

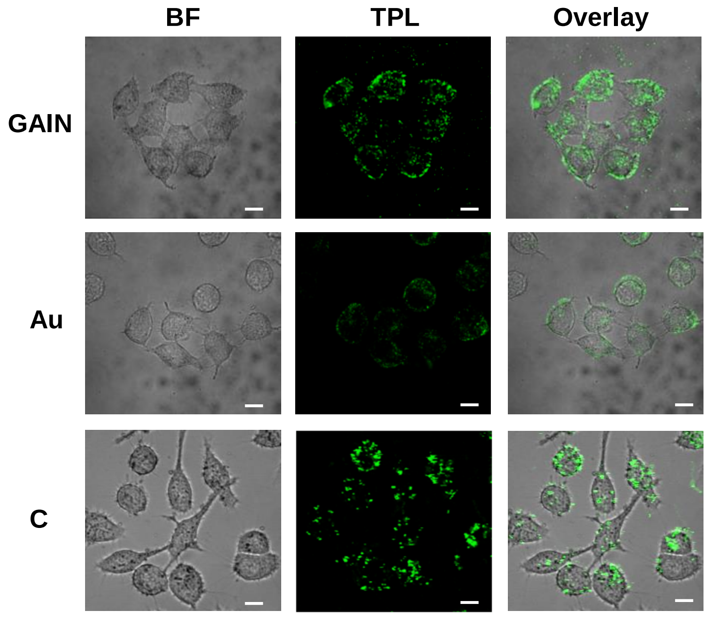

Figure S4. Two-photon microscopy characterization of MFC-7 cells stained with GIAN, Au nanoparticles, and hollow carbon capsulates, respectively. Scale bar: 10 μm.

Figure S5

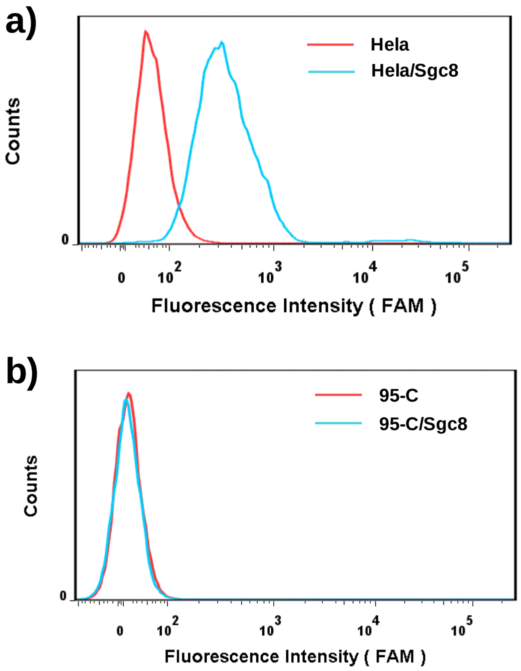

Figure S5. Flow cytometry analysis of the Sgc8 aptamer binding to HeLa (a) and 95-C (b) cells. Sgc8 aptamers were labeled with FAM dyes.

Figure S6

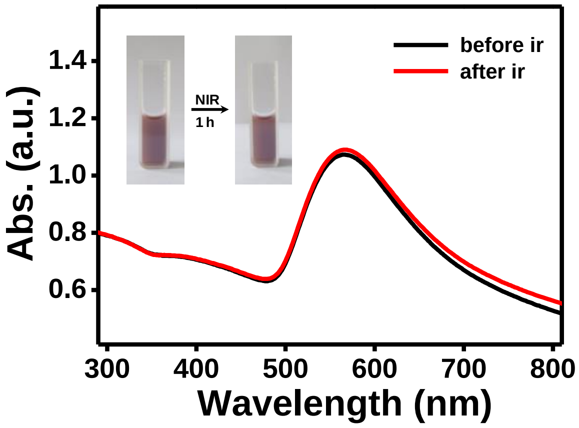

Figure S6. UV-Vis spectra of GIAN suspension before and after 808 nm laser irradiation for 1 hour. The inset shows the digital photo of GIAN suspension before and after irradiation.
